# Integrating the Public in Mosquito Management: Active Education by Community Peers Can Lead to Significant Reduction in Peridomestic Container Mosquito Habitats

**DOI:** 10.1371/journal.pone.0108504

**Published:** 2014-09-25

**Authors:** Kristen Healy, George Hamilton, Taryn Crepeau, Sean Healy, Isik Unlu, Ary Farajollahi, Dina M. Fonseca

**Affiliations:** 1 Center for Vector Biology, Department of Entomology, Rutgers University, New Brunswick, New Jersey, United States of America; 2 Monmouth County Mosquito Extermination Commission, Eatontown, New Jersey, United States of America; 3 Mercer County Mosquito Commission, West Trenton, New Jersey, United States of America; University of Queensland & CSIRO Biosecurity Flagship, Australia

## Abstract

Mosquito species that utilize peridomestic containers for immature development are commonly aggressive human biters, and because they often reach high abundance, create significant nuisance. One of these species, the Asian tiger mosquito *Aedes albopictus,* is an important vector of emerging infectious diseases, such as dengue, chikungunya, and Zika fevers. Integrated mosquito management (IMM) of *Ae. albopictus* is particularly difficult because it requires access to private yards in urban and suburban residences. It has become apparent that in the event of a public health concern due to this species, homeowners will have to be active participants in the control process by reducing mosquito habitats in their properties, an activity known as source reduction. However, limited attempts at quantifying the effect of source reduction by homeowners have had mixed results. Of note, many mosquito control programs in the US have some form of education outreach, however the primary approach is often passive focusing on the distribution of education materials as flyers. In 2010, we evaluated the use of active community peer education in a source reduction program, using AmeriCorps volunteers. The volunteers were mobilized over a 4-week period, in two areas with approximately 1,000 residences each in urban Mercer and suburban Monmouth counties in New Jersey, USA. The volunteers were first provided training on peridomestic mosquitoes and on basic approaches to reducing the number of container habitats for mosquito larvae in backyards. Within the two treatment areas the volunteers successfully engaged 758 separate homes. Repeated measures analysis of variance showed a significant reduction in container habitats in the sites where the volunteers actively engaged the community compared to untreated control areas in both counties. Our results suggest that active education using community peer educators can be an effective means of source reduction, and a critical tool in the arsenal against peridomestic mosquitoes.

## Introduction

Container-inhabiting mosquitoes, such as *Aedes albopictus* (Skuse) and *Aedes aegypti* L., are serious nuisance pests and vectors of disease-causing pathogens to humans. Because of their close contact with human populations, and their vector competence for chikungunya, dengue, and other arboviruses [1], they are important targets for control. *Aedes albopictus*, which is also commonly referred to as the Asian tiger mosquito, oviposits in residential containers, which can be numerous and hard to detect and treat [2]. Therefore, control of mosquito larvae, which forms the basis of peridomestic Integrated Mosquito Management (IMM), is often difficult over large areas [3]. Source reduction, the removal of habitat used by mosquito larvae, can greatly affect the distribution of mosquito larvae in a neighborhood [4], by limiting the amount of habitats for ovipositing mosquitoes and can lead to significantly lower number of adult mosquitoes [3]. In the United States, where this study was conducted, the most common container habitats in residential areas are those classified as non-disposable, such as bird baths, trash cans, planters, plant dishes, and toys [2], [5]. This is similar to studies in other countries that have shown water holding containers, such as flower vases [6] and water pots [7] are important habitats for *Aedes aegypti.* These types of habitats can be difficult to eliminate, since they require public knowledge and engagement in control efforts. Without the public’s involvement in evaluating and managing standing water habitats weekly, these containers will continue to be potential oviposition sites for mosquitoes throughout the course of a season [2], [4]. Therefore, public educational campaigns may have beneficial effects on vector control within those communities [3], [8], by teaching the public how to maintain or eliminate these types of habitats to prevent mosquitoes from completing their development. Community participation is an essential and cost-effective means of reducing container mosquitoes but requires community ownership to achieve sustainability [9], [10]. When successful, the investment in community participation can significantly impact populations of container-inhabiting mosquitoes through source reduction of container habitats, although the impact on adult populations is not always immediately observed [11].

The goal of community education projects is to educate the residents of an area as a whole and involve local leaders, in an attempt to help the community become self-reliant. Community organization and participation can be used as a means of intensifying source reduction efforts. However, for community participation programs to be effective, they must clearly define the participation roles, as well as optimally involve community leaders and/or local government [11]. Soedarmo [12] used community participation in Indonesia as a method of controlling dengue hemorrhagic fever outbreaks. In their program, they trained community members, who were each responsible for providing health education to 500 homes. After 3 months, they found a 56% reduction in larval indices, which measure the prevalence of larvae in containers and levels of infestation [12]. A similar study by Phanthumachinda et al. [13] in Thailand, showed a significant reduction in the Breteau index, a standard measure of mosquito infestation [14], where community volunteers were trained in mosquito control. In a study in Texas, Warner et al. [15] found that high school students were an excellent resource for teaching members of the community regarding health behaviors. One caveat to source reduction programs is that they do not always reduce the risk of disease transmission [16]. However, given that these mosquitoes are important nuisance species, these programs can also benefit the public through reduction of habitats, potential reduction in biting adults [3], and through promoting mosquito control programs in an area.

AmeriCorps is a US national organization that provides service throughout the United States in issues relating to disasters, economy, environment, health, and education [17]. AmeriCorps is part of the Corporation for National and Community Service, whose mission is to improve lives, strengthen communities, and foster civic engagement through service and volunteering [17]. The AmeriCorps National Civilian Community Corps recruits team members aged 18 to 24, who are placed into teams of 8 to 12 other individuals. Teams are then assigned to community-based projects throughout the country.

In 2009 we utilized focus groups of our target audience to help in the development of educational materials, which focused on the message of removing standing water from backyards [5]. Educational brochures and other passive forms of media were then distributed to all members of communities in two sites in New Jersey [3], [5]. The goal of our passive education program was to reduce populations of Asian tiger mosquitoes that use peridomestic containers, and were creating nuisance concerns in the areas. Although our education efforts had a significant impact, it was of limited magnitude (<20% reduction in container habitats in the educational study areas) and we were unable to observe a significant reduction in the numbers of containers when compared to control sites not receiving education [3], [5]. In retrospect we found that a high proportion of residents had education levels at or below the high school level so that providing just reading materials was not the most appropriate strategy for our target audience [5]. Therefore in 2010 we decided to utilize an active community organization public health education approach, where members of the community were approached by peer-volunteers that provided verbal information about public health threats regarding mosquitoes and mosquito-borne diseases. This also involved active demonstrations of ways to minimize the occurrence of peridomestic mosquitoes. We employed a door-to-door campaign, where a large proportion of the community was actively involved in the educational process. We also included additional community events to help increase source reduction efforts.

## Materials and Methods

### Ethics statement

No specific permits were required for collection of field specimens, which were performed in urban and suburban backyards in the US states of New Jersey, Pennsylvania, and Florida with homeowners assent by professional county mosquito control personnel. These studies did not involve endangered or protected species.

### Volunteer recruitment and training

Active education was conducted using AmeriCorps NCCC volunteers, Badger 7 team, as community peer educators. The AmeriCorps team consisted of a team leader, two media representatives, two service-learning initiators, two project outreach liaisons, two corps ambassador program representatives, and a team trainer. Although all team members served the same function while performing door-to-door active education, they were required by AmeriCorps to work on a project related to their team role. Therefore, the AmeriCorps team initiated additional media releases and volunteer opportunities within the communities, which directly benefited our program. From June 21^st^ to August 27^th^ of 2010, the team of AmeriCorps volunteers was deployed to our public health education campaign. During the first week of the project, AmeriCorps members were trained in mosquito biology and source reduction of containers. They were asked to work through various problems and scenarios. Scenarios included images and photographs of backyard habitats, as well as role-playing techniques in simulated backyard habitats. Volunteers were trained to make positive comments in each home, regarding things that homeowners were doing correctly to reduce mosquito habitats. Volunteers were also trained to promote and encourage source reduction by the residents, but not to conduct source reduction themselves.

### Community and active education

Community education occurred during eight weeks in June, July and August of 2010 (June 28^th^ to August 20^th^). The community education involved (1) active education, (2) community presentations, (3) tire-pick up days, (4) trash can drilling days, and (5) media releases. Educational events occurred in two municipalities that had been the focus of several years of control efforts for Asian tiger mosquitoes [18], [3]. These educational treatment sites consisted of a 1,442 parcel area of Cliffwood Beach, New Jersey, and a 1,251 parcel area in Trenton, New Jersey. A parcel consists of a residence and surrounding yard.

Active education was the primary means of education, which involved actively walking around the front and back yard with the resident, describing current and potential mosquito habitats. Volunteers were paired in four to five teams of two, with each team given a daily list of approximately 18 homes to visit. In the interest of volunteer comfort and safety, all teams conducted active education within nearby blocks in the same neighborhoods. After the primary round of active education, a second round was conducted the following week to target residents not home during the initial round of active education. The active educational events were scheduled later in the day during the second round to accommodate those residents that may be coming home from work at that time. To supplement the education, each team was provided educational materials, including brochures, door hangers, mosquito magazines, and build-your-own mosquito activity kits. These materials, which were developed for the prior year’s passive education intervention [5], were not just handed out but enabled the teams to answer questions better, to show the residents pictures of mosquitoes, and to point out other potential types of mosquito producing habitats.

Upon arrival at a home, the team members asked if they could provide free training to residents on how to reduce mosquitoes in their backyards. For those residents willing to be educated, the team would walk around the yard with the resident, describing ways in which they could reduce mosquito habitats in their backyard. Volunteers were asked to always point out one thing the resident was doing correctly. The volunteers also answered questions regarding mosquitoes, and relayed service requests and questions back to the local mosquito control program. When a resident was not home or not willing to be engaged, information on how to reschedule a site visit as well as other events within the community was left at the residence. We left this information in plastic bags hanging from the residence’s front door-handle. This type of information dissemination is commonly referred to as “door-hangers”. Door-hangers were also given to residents we spoke to, since it was our primary means of informing residents about the dates and types of other educational events in the community.

For each house visited, the team members filled out a form describing whether the resident was home, containers observed, the type of activities and level of education performed, and other observations. A residence was only considered “educated” if they actively walked around the property with the team members. Politely accepting a brochure did not constitute being educated. At the end of the day, the teams were asked to fill out a summary form of their activities. The summary form included the number of homes visited, number of abandoned homes, number of homeowners educated, not home, or refusing education, languages other than English, reasons for refusing education, types of containers at the site, service requests for mosquito control, and other activities associated with mosquito reduction (such as collecting tires or drilling drainage holes in trash cans).

### Community events

In addition to door-to-door active education, we conducted several community educational events in both treatment areas. In Monmouth County we left door-hangers advertising free home consultations, community workshops, and tire pick up days. The door hanger was provided to residents whether or not they were home during the active educational events, so all residents would be aware of the date and time of events. The free home consultations included a phone number to call to schedule an expert to inspect the house and show the resident how to reduce mosquitoes. The community workshops included two single hour events where residents could learn how to protect themselves from mosquitoes in their backyard. For the tire-pick up days, residents were asked to leave out their unwanted tires on the curb in front of their house for pick up. At the end of the study, the advertisement for the tire disposal was expanded to other areas in the bay shore of Monmouth County, New Jersey. In Mercer County, we used door-hangers to advertise free home consultations, tire pick up days, and trash can drilling days. For the trash can drilling days, residents were asked to leave their trash cans out at the curb, so volunteers could drill drainage holes in the bottom.

### Container surveys

Container surveys were conducted before the educational events (June and July), after education (August), and two to three months after treatment (October and November) using methodologies and treatment and control sites previously described [5]. Each treatment site receiving education was compared to a demographically similar site that received no education (the control site). In Monmouth County, NJ, the control sites were located in Union Beach Borough, and in Mercer County, the control sites were located in Hamilton. These sites were similar in parcel size and demographics to the treatment sites in Monmouth County (Cliffwood Beach), and Mercer County (Trenton) [18]. Container surveys were conducted by manually counting every container, water-filled container (wet), containers infested with larvae, and containers infested with pupae at each surveyed home. Survey teams would walk the entire property (front, back, and side yards) inspecting all potential container habitats. Containers were defined as anything natural or manmade whose shape and structure allows it to collect and hold water for 2 or more days. We also included a category called “managed containers” (a container that has been deliberately turned upside down, covered, chlorinated, aerated, or maintained in a way that would prevent the collection of water or the production of mosquitoes). An “unmanaged container” was any container that fit the definition of “container” above, and was not being “managed” as described above. Since we were simply looking at behavior change (measured by reduction of habitats), we did not identify larvae or pupae to species. At each home, a spread sheet was used to record the address, date, time of survey, container type, number of containers, number of water-filled containers, and those infested with larvae and/or pupae. All containers, larvae, and pupae were left undisturbed during the container surveys, as not to affect future survey results. We did not collect samples or identify the larvae or pupae to species. In Monmouth County, approximately 50 homes each were selected in the treatment and control sites. In Mercer County, approximately 75 homes each were selected in the treatment and control sites. Homes were selected using Geographic Information System (GIS) technology, using grids to separate out the treatment and control areas into 50 (Monmouth) or 75 (Mercer) zones. The home closest to the center of each grid, when permission was granted, was selected as our survey location. Due to several homes opting out of the study, the final data include 47 treatment and 45 control sites in Monmouth County, and 64 treatment and 64 control sites in Mercer County.

### Analyses

The number of unmanaged containers per home in the treatment and control areas where the AmeriCorps and community events were deployed was compared with those in the paired untreated control sites using a repeated measures analysis of variance in SPSS software (IBM, Armonk, NY, USA). This analysis allowed us to examine the change in the homeowner’s behavior over time (source reduction behavior) in residences receiving education versus those not receiving education. The Breteau index (number of water-filled containers containing larvae and/or pupae per 100 homes) and house index (percentage of homes infested with larvae/pupae) were calculated for each site during each sampling period [14]. The Breteau index is an internationally recognized index of container-inhabiting mosquito populations, which allowed us an additional measure of suitable habitats in the treatment and control sites. We were not attempting to estimate disease transmission risk using these indices. The number of positive (contains larvae and/or pupae) and negative containers per treatment area were compared using Chi Square analysis (SPSS). We performed a spatial analysis, where total number of unmanaged containers for each sampled residence were plotted using ArcMAP GIS (North American Datum 1983). For each site and month, maps were created using inverse distance weighted (IDW) mapping tools. The parameters for the IDW included using a variable search radius, references the 12 closest points, and weighting those points closest heavier than those further away (IDW squared). The color reference scales for map interpolations were standardized to the same scale, and compared by looking at overall trends in the dataset. These interpolations allowed us to ascertain if the numbers of containers per home were uniformly distributed or were influenced by homes with large numbers of containers per yard. We calculated the difference between pre-educational survey interpolations and interpolations of surveys immediately following education using ArcMAP’s spatial analyst math tools. This analysis allowed us to examine the overall change in behavior spatially (positive or negative) regardless of the variation in the number of containers per home. Difference maps were color coded either black (increase in containers) or grey (decrease or no change in containers). The resulting image was analyzed in ImageJ by calculating out the total black and grey geometric areas within each image. A chi square analysis of the resulting geometric area was performed to assess whether observed behavioral changes between residents pre-education and immediately following education were independent of educational events within the community.

## Results

### Monmouth county

**Figure 1:**
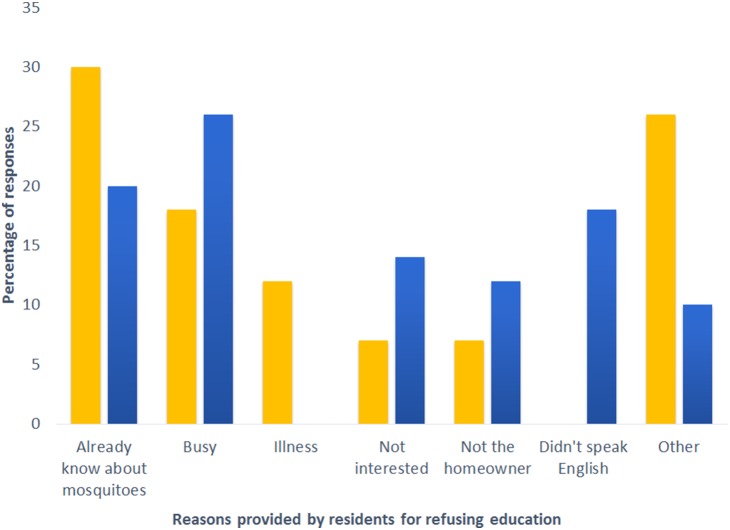
Reasons provided by residents within the educational treatment site for not wanting to be actively educated by the community peer educators. Responses from residents in Monmouth County are represented in yellow, and those from Mercer County are represented in blue.

Active education in Monmouth County occurred during the four-week period from June 28^th^ to July 23^rd^. Of the 1,442 parcels in the Monmouth County treatment area, 1,050 contained occupied homes. The remaining parcels consisted of open areas, parks, businesses, and abandoned homes. It was estimated that only 23 homes within the treatment area were abandoned (less than 1%). Of the 1,050 occupied homes, 544 homes (51.8%) contained at least one individual at home during our educational interventions. Of these, 394 were willing to walk around the yard and be “actively” educated by the volunteers, while 150 refused. The primary reason (30%) for refusing education was that they “already knew about mosquitoes” (Figure 1).

Events in Monmouth County included a full week of tire pick up days, one community presentation, and advertisements for free consultations (for those individuals who were not home during the education). A total of 97 tires were picked up from the front curbs of 12 homes during the tire pick up days. The number of tires per curb (mean = 8.8) ranged from 1 to 22 tires. Of the 12 homes participating in the tire pick up days, tires were received from homes that were actively educated (50%), informed from a door hanger only (30%), and from individuals refusing education (20%). After the initial tire pick up days, the tire drop off was advertised to the rest of the county. An additional 730 used tires were picked up after advertising countywide. No one from the educational treatment site attended the community presentations or requested free at home consultations.

**Figure 2:**
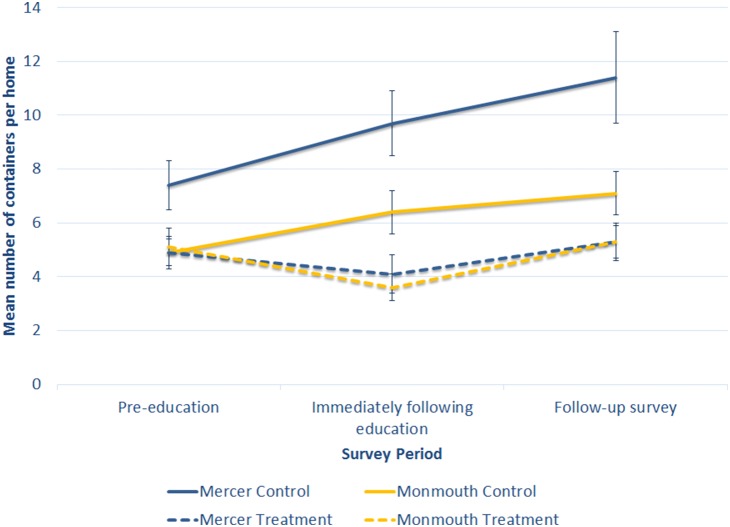
Mean number of unmanaged containers per homes (± SE) in sites receiving active education (Treatment site), versus those not receiving education (Control site). The Mercer County data is represented in blue, and the Monmouth County data represented in yellow. Treatment sites are represented by the dashed lines, and control sites by the solid lines.

Container surveys were conducted in 47 treatment parcels (parcels within the active education community) and compared to results from 45 control parcels (parcels within the community not being educated). Surveys were conducted prior to active and community education (June 23^rd^ to the 25^th^), immediately following education (August 23^rd^ to the 25^th^), and 15 weeks (3 months) after educational events (November 11^th^ to the 15^th^). In the treatment site, the mean number of unmanaged containers per home was reduced from 5.1± 0.7 (SE) containers per home prior to education to 3.6±0.5 (SE) containers per home following education. However, this decrease was not sustained, and increased back three months after education to 5.3±0.6 (SE) containers per home. In the control site, the number of containers per home continually increased from 4.9±0.6 (SE) to 6.4±0.8 (SE) to 7.1±0.8 (SE) containers per home. Repeated measures analysis (F = 3.7, P = 0.029) showed a significant reduction of container habitats in the treatment site, which remained lower than the control site even at the three months follow-up survey (Figure 2).

Of the total unmanaged containers sampled in the treatment site, only 4 contained larvae or pupae during the pre-education inspection, and did not differ (X^2^ = 1.09, P = 0.3) from our control site, which had 8 larvae or pupae filled containers (Table 1). During our three month follow up inspection, only 7 containers had larvae and pupae in the education site, compared to 29 in our control sites (X^2^ = 8.3, P = 0.004). Positive containers were not confined to a single residence as was indicated by our house indices (Table 1). The resulting Breteau indices were 14.9 in the treatment site, compared to 63.0 in our control site (Table 1). Infested container habitats in the control site included buckets (24.1% of the total infested containers counted), children’s play pools (24.1%), toys (13.8%), planter dishes (13.8%), wheelbarrows (6.9%), pool covers (6.9%), hampers (3.4%), tires (3.4%), and an ornamental pond (3.4%). In the education site, infested habitats included a single pool cover, rain barrel, planter dish, ramp, tarp, children’s play pool, and two buckets.

**Table 1:** Breteau index (number of containers infested with larvae and/or pupae per 100 houses) and House index (percentage of homes infested with larvae and/or pupae) for sites with community and active education (treatment) and those not receiving education (control sites) in Monmouth and Mercer Counties.

		Breteau index (house index)		Number of infested containers (total number of containers observed)			
County	Event	Treatment area	Control area	Treatment area	Control area	Chi square value	p-value
**Monmouth County**	Pre-education	8.5 (8.5)	17.3 (15.6)	4 (242)	8 (220)	1.09	0.3
**Monmouth County**	November follow up	14.9 (14.9)	63.0 (20.0)	7 (250)	29 (319)	8.33	0.004*
**Mercer County**	Pre-education	4.7 (3.1)	3.1 (3.1)	3 (311)	2 (472)	0.23	0.63
**Mercer County**	October follow up	3.1 (3.1)	0.0 (0.0)	2 (337)	0 (731)	1.75	0.19

Data are included for pre-educational events, and those at the end of the study (October and November follow-up surveys). The total containers includes all unmanaged containers (both dry and water filled) in the treatment and control areas. Chi-square values (comparing infested containers in the treatment in control sites) that are statistically significant are indicated with an asterisk.

**Figure 3:**
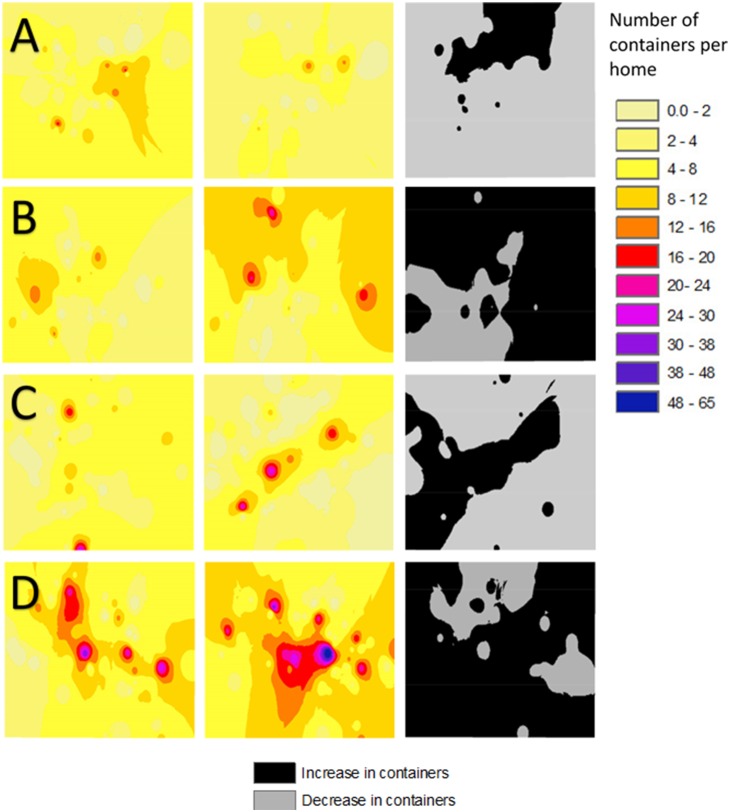
Spatial distributions of the number of container habitats per home sampled in sites receiving education (treatment sites) and those not receiving education (control sites). The columns represent the number of containers before education, (1^st^ column) immediately following education (2^nd^ column) and the difference between the two (3^rd^ column, in black & grey). In homes with no change in container number, they were classified along with a decrease in containers. The rows summarize Monmouth county treatment site (A), Monmouth county control site (B), Mercer county treatment site (C), and Mercer county control site (D).

Spatial analysis allowed us to examine spatial patterns of source reduction behavior (Figure 3). In Monmouth County, an examination of before and after maps, showed that treatment sites exhibited more source reduction behavior, whereas control sites had a greater amount of container habitat increase. Results of the ImageJ analysis, showed that the Monmouth county treatment site had a geometric area of 83.5% exhibiting source reduction behavior (Figure 3C). This is contrast to the control site (that did not receive education) that had a geometric area of 25.9% exhibiting source reduction behavior (Figure 3F). Chi square analysis of ImageJ results (X^2^ = 66.28, P<0.001) indicated that source reduction behavior was different between treatment and control sites.

### Mercer county

Active education in Mercer County took place from July 26^th^ to August 20^th^. Of the 1,251 parcels in the Mercer County treatment area, 962 contained occupied homes. The remaining parcels consisted of open areas, parks, businesses, and abandoned homes. It was estimated that 126 homes within the treatment area were considered abandoned (12%). Of the 962 occupied homes, 507 homes (52.7%) contained at least one individual at home during our educational interventions. Of these, 364 were willing to walk around the yard with the educators, while 143 refused. The primary reason (26%) for not wanting to be educated was that they were “too busy” (Figure 1).

Events that were performed in Mercer County included tire pick up days, trash can drilling days, and advertisement for free consultations (for those individuals who were not home during the education). Tires were continually picked up by other control interventions but were not quantified. A total of 48 trash cans were drilled, as part of the advertised trash can drilling days. This included individuals that were actively educated (40%) and individuals that were informed from a door hanger only (60%). There were no requests for the free home consultations.

Container surveys were conducted in 64 treatment parcels (parcels within the active education community) and compared to results from 64 control parcels (parcels within the community not being educated). Surveys were conducted prior to active and community education (July 9^th^ to the 14^th^), immediately following education (August 23^rd^ to the 25^th^), and 6 weeks after educational events (October 5^th^ to the 13^th^). In the treatment site, the mean number of unmanaged containers per home slightly decreased from 4.9±0.5 (SE) prior to education to 4.1±0.7 (SE) after education. However, this was not sustained, and increased to 5.3±0.7 (SE) in the follow-up survey. In the site not receiving education, the number of containers per home continually increased from 7.4±0.9 (SE) to 9.7±1.2 (SE) to 11.4±1.7 (SE). Repeated measures analysis (F = 3.9, P = 0.023) showed a significant reduction of container habitats in the treatment site compared to the increase in containers in the control site (Figure 3).

Of the total unmanaged containers sampled in the treatment site, only 3 contained larvae or pupae during the pre-education inspection, and did not differ (X^2^ = 0.23, P = 0.63) from our control site, which had 2 larvae or pupae insested containers (Table 1). During our 6 week follow up inspection, only 2 containers had larvae and pupae in the education site, compared to 0 in our control sites (X^2^ = 1.75, P = 0.19). Positive containers were not confined to a single residence as was indicated by our house indices (Table 1). The resulting Breteau indices were 3.1 in the treatment site, compared to 0.0 in our control site (Table 1). Infested container habitats in the education site included a bucket and a rolling stand. There were no larvae/pupae infested containers in the control site during the final container survey.

Spatial analysis allowed us to examine spatial patterns of source reduction behavior (Figure 3). In Mercer County, an examination of before and after maps, showed that treatment sites exhibited more source reduction behavior, whereas control sites had a greater increase in container habitats. Results of the ImageJ analysis, showed that the Mercer county treatment site had a geometric area of 68.2% exhibiting source reduction behavior (Figure 3I), whereas the Mercer county control site had a geometric area of 23.0% exhibiting source reduction behavior (Figure 3L). Chi square analysis of ImageJ results (X^2^ = 40.8, P<0.001) indicated that source reduction behavior was different between treatment and control sites.

## Discussion

The goal of this study was to test the feasibility of using a community-based education program for mosquito control agencies locally and nationally in the USA. We hope that our study will provide a foundation or a model for local, state, and national organizations faced with implementing an emergency response to mosquitoes and mosquito-borne disease epidemics. The use of volunteers during these emergencies can help facilitate the interaction with the public, to ensure that the public is informed regarding ways to minimize risk.

Using active education, we were able to observe a 22.6% reduction in container habitats in the communities being educated (treatment site), compared to a 32.3% increase in the sites not receiving education (control sites). Interestingly, trends in container abundance were similar for both counties. Prior to this study, we had evaluated passive educational techniques in promoting source reduction behavior [5]. Although we observed some source reduction behavior during the passive education study, the educational study sites were not statistically different from the control sites, and showed the same trends throughout the season [5]. These results were similar to other studies that had relied on passive forms of education [19], [20]. As with the 2010 results, we observed similar trends in both counties in 2009. During our previous study in 2009, we observed a willingness to help empty standing water on their property, when we had an interaction with the homeowner. In addition, we felt the presence of mosquito personnel counting containers could have indirectly motivated residents to conduct source reduction [5]. This led us to hypothesize during our previous study that an active education campaign could better promote source reduction behavior in the community. We feel our current results support this hypothesis, given the significant differences in the repeated measures analysis through active education, compared to the lack of a significance using passive education [5].

Of the 1,051 residents that were home during the educational program, 72% were willing to walk around their back yard with our community peer educators. By itself, this finding underscores the impact of mosquitoes on our communities, as several studies have shown that *Ae. albopictus* influences the quality of life and outdoor activities of both adults and children [9], [21], [22]. Halasa et al. [23] conducted a survey of individuals within our communities and found that mosquitoes prevented 74% of the respondents of the survey from enjoying outdoor activities. Even without the real threat of disease, biting pressure caused by mosquitoes appears to provide enough motivation within the community for individuals to actively get involved in their control. Indeed, Dickinson and Paskewitz [24] showed that nuisance is a more important motivator than disease risk, a result that may seem counterintuitive but should be garnered by public health officials and mosquito control programs.

Although 28% of residents that were home when the volunteers arrived were not willing to be educated, we found that several of these residents still participated in community events. Over 20% or residents participating in the tire pick up days had refused education from our community peer educators. Tires are an important mosquito habitat for *Ae. albopictus,* and comprise a large percentage of the container habitats in our study areas [25]. Therefore, targeted interventions towards specific container types can provide an important means of reducing primary habitats from within a community.

Our study showed a significant reduction of container habitats in both treatment areas receiving education versus the control sites not receiving education. The reduction was not sustained, since we saw some increase in containers over time. However the percent increase in container habitats in the education sites over the entire season (5.6%) was far less of that in the control sites (52.1%). In addition, the increase in container habitats needs to be assessed in the context of the entire season. In the warm and humid summers in New Jersey, the number of water-filled containers increases over the summer as residents spend more time outdoors and rain events fill them with water. This increase is readily exploited by *Ae. albopictus* females that maximize their offspring’s fitness by spreading their eggs across many containers [26]. Overall, the sites where the community was engaged twice during the season (in July and middle August) still maintained a lower number of sources of peridomestic mosquitoes as indicated by the overall number of container habitats, a trend that was observed in both counties.

In addition to habitat reduction, we saw a decrease in larvae or pupae infested containers in the Monmouth county treatment parcels compared to the control. However, we did not see the same trend in the Mercer county parcels. The low Breteau indices in Mercer likely reflect the fact that 2010 was a record hot and dry year in New Jersey [27], which depressed the population of *Ae. albopictus* significantly in the very urban and therefore warmer Trenton site [28] but not in Monmouth. In 2010, August and September were relatively dry months, which could have reduced availability of habitat for container mosquitoes. In Mercer county, very few containers (1.7% of all sampled) contained water during the final survey. This was very different to what was seen in Monmouth County, where 30.4% of all sampled containers in the final survey contained water. Therefore, the two counties should be evaluated independently when considering larval indices.

Throughout the study, we attempted to evaluate the performance of the teams in educating homeowners by having one of the co-authors visit a property as it was visited by one of the teams. Each day, a different team was evaluated to assess their quality. We found all teams were always polite and did an excellent job in pointing out things homeowners were doing correctly. However, since the volunteers were not mosquito biologists or control experts, there were occasional instances where a fully or partially inconspicuous container habitat would go unnoticed. The quality of education may have therefore varied from team to team and throughout the study. However, our expectation is that once a homeowner becomes educated regarding mosquito habitats, they can sustain source reduction efforts by continually finding sources of habitats on their own property. This is essential, since source reduction efforts done solely by mosquito control personnel, in the absence of sustained effort, can result in the repopulation of key mosquito habitats [2].

Although our program evaluated the use of AmeriCorps volunteers as part of an area-wide mosquito management program, there are other volunteer organizations that could participate in mosquito control activities. These include the Medical Reserve Corps (MRC), Boy Scouts of America, church groups, citizen scientists, community volunteers, and agricultural extension programs. The Medical Reserve Corps has volunteers in every state, and most often in the most populated counties within those states. The mission of the MRC is to engage volunteers to strengthen public health, emergency response, and community resiliency [29]. Therefore, MRC already functions in providing emergency public health functions, and could easily be incorporated into public health responses regarding vector-borne diseases. In addition, extension agents associated with state universities in the US are often trained to assist in outreach efforts and can be found in all states and territories in the United States [30].

The Breteau index, as well as other larval indices, are traditional tools that have been used to estimate the local vector populations within a community [14]. However, there are shortcomings to these indices, especially as they relate to disease transmission [14], [16]. Our results are consistent with several other studies that have shown stable or decreasing Breteau indices in community-based programs compared to increasing indices in control sites not receiving education [31], [32]. Although a reduction in indices does not usually relate to a reduction in vector-borne diseases, such as Dengue [16], the goal of this study was not to reduce or estimate transmission risk, but to examine several measures to estimate source reduction behavior within the community. Given that our public education message was to reduce container habitats, we felt that container surveys were a more reliable estimate of source reduction behavior, given that education does not always result in immediate reduction in adult mosquito populations [11].

Numerous studies have shown the value of community participation in source reduction of container mosquitoes [10], [31], [32], [33]. Community-based programs targeting species, such as *Aedes aegypti*, have not only been effective in reducing container habitats, but also in reducing house, container, and Breteau indices [7], [31], [32]. There is also the added benefit of increasing the knowledge of the members of the community in recognition of habitats, transmission of disease, and personal protection [31], [32]. These programs can also be effective in changing the behavior of individuals by reducing larval habitats [31]. Although source reduction alone cannot effectively control container mosquitoes [2], these programs can be essential to help reduce potential habitats and sustain source reduction efforts within a community. Although programs that focus on behavior changes through public messages are often short term [34], future community based programs must promote the public to be self-sufficient [33]. Therefore, every effort should be made to involve the community when developing source reduction campaigns [10], [33]. This can also be accomplished by better understanding the sociology and determinants within the community, as these can greatly affect the habitat types and behaviors within a specified community [6].

The goal of this study was to evaluate the use of community peer educators in promoting reduction of mosquito habitats for mosquitoes, especially *Ae. albopictus,* an important vector of dengue, chikungunya and Zika viruses. Since *Culex* vectors of West Nile virus also utilize container habitats, there is an added benefit for the control of these important species as well. Studies have shown that the most effective education campaigns are those where the community has ownership of the program [10]. Although public health educational campaigns do not always have an immediate effect on the population of mosquitoes [11], community participation can help reduce mosquito habitats of important vectors, while developing long term, low-cost, sustainable programs [10], [35]. In the event of a public health emergency, such as an outbreak of a vector-borne disease, community peer educators can both help in the reduction of vector habitats, as well as provide reassurance back to the community regarding mosquito control programs in the area.
